# Proteome-Wide Screening Reveals Immunodominance in the CD8 T Cell Response against Classical Swine Fever Virus with Antigen-Specificity Dependent on MHC Class I Haplotype Expression

**DOI:** 10.1371/journal.pone.0084246

**Published:** 2013-12-23

**Authors:** Giulia Franzoni, Nitin V. Kurkure, Sabine E. Essler, Miriam Pedrera, Helen E. Everett, Kikki B. Bodman-Smith, Helen R. Crooke, Simon P. Graham

**Affiliations:** 1 Virology Department, Animal Health and Veterinary Laboratories Agency, Addlestone, United Kingdom; 2 Department of Microbial & Cellular Sciences, University of Surrey, Guildford, United Kingdom; 3 Nagpur Veterinary College, Maharashtra Animal & Fishery Sciences University, Nagpur, India; 4 Department of Pathobiology, University of Veterinary Medicine Vienna, Vienna, Austria; The Ohio State University, United States of America

## Abstract

Vaccination with live attenuated classical swine fever virus (CSFV) vaccines induces a rapid onset of protection which has been associated with virus-specific CD8 T cell IFN-γ responses. In this study, we assessed the specificity of this response, by screening a peptide library spanning the CSFV C-strain vaccine polyprotein to identify and characterise CD8 T cell epitopes. Synthetic peptides were pooled to represent each of the 12 CSFV proteins and used to stimulate PBMC from four pigs rendered immune to CSFV by C-strain vaccination and subsequently challenged with the virulent Brescia strain. Significant IFN-γ expression by CD8 T cells, assessed by flow cytometry, was induced by peptide pools representing the core, E2, NS2, NS3 and NS5A proteins. Dissection of these antigenic peptide pools indicated that, in each instance, a single discrete antigenic peptide or pair of overlapping peptides was responsible for the IFN-γ induction. Screening and titration of antigenic peptides or truncated derivatives identified the following antigenic regions: core_241–255_ PESRKKLEKALLAWA and NS3_1902–1912_ VEYSFIFLDEY, or minimal length antigenic peptides: E2_996–1003_ YEPRDSYF, NS2_1223–1230_ STVTGIFL and NS5A_3070–3078_ RVDNALLKF. The epitopes are highly conserved across CSFV strains and variable sequence divergence was observed with related pestiviruses. Characterisation of epitope-specific CD8 T cells revealed evidence of cytotoxicity, as determined by CD107a mobilisation, and a significant proportion expressed TNF-α in addition to IFN-γ. Finally, the variability in the antigen-specificity of these immunodominant CD8 T cell responses was confirmed to be associated with expression of distinct MHC class I haplotypes. Moreover, recognition of NS2_1223–1230_ STVTGIFL and NS3_1902–1912_ VEYSFIFLDEY by a larger group of C-strain vaccinated animals showed that these peptides could be restricted by additional haplotypes. Thus the antigenic regions and epitopes identified represent attractive targets for evaluation of their vaccine potential against CSFV.

## Introduction

Classical swine fever (CSF) is a severe and often lethal viral disease of domestic pigs and wild boars. The aetiological agent is classical swine fever virus (CSFV), a small, enveloped, positive-sense, single-stranded RNA virus belonging to the pestivirus genus of the *Flaviviridae* family [1], [2]. The disease is endemic in South East Asia, parts of Central and South America and the Russian Federation. Despite the stringent controls adopted in the EU, the virus continues to be an epizootic threat with recent outbreaks in Lithuania (2009 and 2011) and Latvia (2012) [3]. CSF is amenable to control by vaccination and live attenuated C-strain vaccines are highly efficacious. However, the inability to differentiate vaccinated animals from those infected with CSFV limits their utility as a control tool in outbreak settings in the EU [4]. Control of CSF outbreaks via a stamping-out policy is expensive, because large numbers of animals have to be culled including those slaughtered pre-emptively. Public resistance against such drastic measures is also growing. As a consequence, there is increased pressure to develop and adopt alternative strategies, like marker vaccines, to aid the control of CSF outbreaks [4].

C-strain vaccine induced IFN-γ responses have been correlated to rapid protection against the disease [5] and CSFV-specific IFN-γ secreting CD8 T cells are detected in the blood early after vaccination [6]. Determining the viral proteins that are the targets of the CD8 T cell response in immune animals would provide an important step towards developing a next generation marker vaccine capable of providing rapid protection against CSFV. CSFV has four structural proteins (the core protein and the envelope glycoproteins Erns, E1 and E2) and eight non-structural proteins (Npro, p7, NS2, NS3, NS4A, NS4B, NS5A, and NS5B) [1]. E2 and NS3 have been described as targets of the T cell response and both proteins induce IFN-γ release [6]–[9] and cytotoxic activity by T cells from vaccinated pigs [7]–[10]. A T cell epitope was identified on NS4 [9] and our group recently reported NS5B as a putative target of IFN-γ secreting T cells from C-strain vaccinated pigs [6]. Epitopes may be located on other viral proteins, since peptides pooled to represent Erns, E1, NS2, NS4B and NS5A were able to induce PBMC proliferation in vaccinated pigs, but their ability to elicit an IFN-γ or cytotoxic response was not tested [9]. Most of these studies utilised inbred homozygous pigs so were focussed on a single haplotype [7], [9], [10] and the phenotype of the responding T cells/MHC restriction was not or only partially characterized [6]–[10].

Knowledge of epitopes within viral proteins that are targeted by CD8 T cell is also necessary to ensure that genetically attenuated or sub-unit DIVA vaccines include these regions. As the major target of neutralizing antibody responses, the structural protein E2 has been used to create subunit or chimeric vaccines [11], [12]. Additional evidence that this protein is also able to target the cellular immune response comes from a recent study which showed that a DNA vaccine expressing E2 induced a cellular immune response, characterized by IFN-γ releasing T cells, before the appearance of neutralizing antibodies [11]. Moreover, a chimeric vaccine CP7_E2alf, where the E2 protein of the CSFV strain Alfort 187 is inserted in the backbone of the bovine viral diarrhoea virus (BVDV) strain CP7, can fully protect pigs from challenge before the appearance of neutralizing antibodies [12]. However, it remains to be determined whether CSFV E2 specific or BVDV cross-reactive antigen specific T cells are involved in mediating this protection.

The ability of subunit vaccines to trigger T cell responses that contribute to protection has been observed in other viruses belonging to the family *Flaviviridae*, such as hepatitis C virus (HCV) [13]-[15] and dengue virus (DENV) [16], [17]. Both a HCV peptide-vaccine, including 5 MHC-class I and 3 MHC class-II-restricted epitopes, a DNA vaccine encoding NS3 and NS4 and an adenovirus-based vaccine expressing NS3 of HCV induce a strong CD8 T cell response in vaccinated individuals [13]–[15]. DNA vaccines based on the NS3 protein from DENV and an adenovirus expressing DENV NS1 induce a peptide-specific IFN-γ response by CD8 T cells from vaccinated mice which correlated with protection [16], [17].

With a view to better defining the protein and epitope targets of the CD8 T cell response in pigs protected against CSF by vaccination with a live-attenuated C-strain vaccine; we screened a peptide library spanning the CSFV proteome. Five T cell antigens and the corresponding antigenic regions/epitopes were identified. The conservation of these peptides across CSFV strains was assessed as were the restricting MHC class I haplotypes and the CSFV-specific T cells responding were phenotypically and functionally characterized.

## Materials and Methods

### Ethics Statement

All work was approved by the Animal Health and Veterinary Laboratories Agency Ethics Committee and all procedures were conducted in accordance with the UK Animals (Scientific Procedures) Act 1986 under project licence permit numbers PPL 70/7403 and PPL 70/6559. Regular observation and completion of clinic scoring sheets was conducted, which informed euthanasia decisions based on a pre-defined humane endpoint (clinical score >15 or if temperature >41°C and more than 2 individual scores (other than the temperature score) have a value of 3) and minimised potential suffering to the animals. None of the animals reported in this study experienced clinical signs necessitating euthanasia. All animals were euthanized on pre-determined days by intramuscular administration of Ketamine/Rompun sedative followed by intravenous administration of 20% sodium pentobarbitone solution.

### Viruses

A commercial live attenuated C-strain CSFV (genotype 1.1, AC Riemser® Schweinepestvakzine, Riemser Arzneimittel AG, Riems, Germany), the highly virulent Alfort-187 (genotype 1.1, AHVLA CSF Reference Laboratory Virus Archive, Addlestone, UK) and Brescia (genotype 1.1, kindly provided by Dr Alexandra Meindl-Böhmer, University of Veterinary Medicine, Hannover, Germany) strains, the moderately virulent UK2000/7.1 (a genotype 2.1 isolate from the UK [18]) and CBR/93 (a genotype 3.3 isolate kindly provided by Dr Sujira Parchariyanon [19]) strains of CSFV were propagated in porcine kidney (PK15) cells maintained in Eagle's Minimum Essential Medium (E-MEM) (Life Technologies, Paisley, UK) with 10% FBS (Autogen Bioclear, Calne, UK) and antibiotics (100 U/ml penicillin, 100 µg/ml streptomycin, both from Life Technologies). CSFV was harvested from cultures after 4 days and TCID_50_ titres were determined according to standard protocols [20]. Mock virus supernatants were prepared in an identical manner from uninfected PK15 cells.

### Vaccination and challenge of pigs with CSFV

#### Experiment 1

An experimental vaccination/challenge study was performed to assess the specificity of CSFV-specific CD8 T cell IFN-γ responses, as previously described [21]. In brief, eleven Large White/Landrace pigs, 6 months of age, were utilized; eight animals were vaccinated 5 days before challenge (day -5) by intramuscular inoculation of 10^5^ TCID_50_ of C-strain CSFV and challenged on day 0 and 28 by intranasal inoculation of 10^5^ and 10^6^ TCID_50_ of CSFV Brescia, respectively (1 ml divided equally between each nostril and administered using a mucosal atomization device MAD-300, Wolfe Tory Medical, Salt Lake City, USA). Three negative control pigs received similar inoculations of mock virus supernatant on each occasion. Virus back titrations of the inocula confirmed the vaccine and challenge doses were those expected.

#### Experiment 2

A second vaccination/challenge study assessed recognition of identified T cell epitopes by additional C-strain vaccinated pigs. Animals vaccinated and challenged in two independent, previously described, experiments [5] were utilised. Large White/Landrace cross male pigs, 9 weeks of age, were vaccinated intramuscularly with 2 ml of reconstituted C-strain vaccine, as described by the manufacturer (Riemser Arzneimittel AG) and after 3 or 5 days were challenged by intranasal inoculation of 10^5^ TCID_50_ CSFV UK2000/7.1 or CBR/93 strains as described above.

### Clinical and haematological methods

Temperatures and clinical scoring were monitored for 7 days before and after each of the CSFV Brescia inoculations for Experiment 1 [21] and from day -5 to 15 days post-challenge for Experiment 2 [5]. Nine parameters relevant for indication of CSF (liveliness, body tension, body shape, breathing, walking, skin, eye/conjunctiva, appetite/leftover at feedings, defecation) were examined and scored as 0 (normal), 1 (slightly altered), 2 (distinct clinical sign) or 3 (CSF signs). A total clinical score for each animal was assigned twice daily and temperatures were monitored by daily rectal thermometer readings [22]. Peripheral blood leukocyte counts were monitored by volumetric flow cytometry during both experiments [22]. In brief, 50 µl EDTA blood was incubated with 5 µl of anti-porcine CD45-FITC monoclonal antibody (mAb) (0.1 mg/ml, K252-1E4, AbD Serotec, Oxford, UK) for 10 minutes at room temperature (RT) in the dark. FACS Lysing solution (945 µl) (BD Biosciences, Oxford, UK) was added for 10 minutes at RT to lyse erythrocytes and fix leukocytes. Cell counts were obtained on a volumetric flow cytometer (MACSQuant Analyzer, Miltenyi Biotec, Bisley, UK) by gating FITC positive events and leukocyte counts per µl/blood were obtained by multiplying the leukocyte density by the dilution factor (x 20).

### Purification and cryopreservation of peripheral blood mononuclear cells (PBMC)

Heparinised venous blood was collected on days -5, 0 and every 7 days until day 56 post-challenge for Experiment 1 and every three days from day -5 to 15 post-challenge for Experiment 2. PBMC were prepared by diluting 20 ml of blood in 10 ml PBS (Life Technologies), layering over 20 ml of Histopaque-1077 (Sigma-Aldrich, Poole, UK) and centrifuging at 1455×*g* for 30 minutes at room temperature (RT), without braking, in a rotating bucket centrifuge. PBMC were aspirated from the plasma-Histopaque-1077 interface and washed three times in PBS by centrifugation at 930×*g* for 5 minutes at 4°C. PBMC were re-suspended in RPMI-1640 medium (Life Technologies) supplemented with 10% FBS, 100 U/ml penicillin and 100 µg/ml streptomycin (cRPMI) and cell densities determined using a volumetric flow cytometer (Miltenyi Biotec) and gating on events with typical forward scatter (FSC) and side scatter (SSC) for PBMC. Cells were used directly for phenotypic/functional analysis or cryopreserved for subsequent analysis.

For cryopreservation, PBMC were adjusted to a density 1–2×10^7^ cells/ml, re-suspended in cold (4°C) 10% DMSO (Sigma-Aldrich) in FBS and transferred to pre-cooled (4°C) labeled cryotubes. These tubes were immediately transferred to pre-cooled (4°C) Cryo 1°C Freezing Container (Nalgene, Fisher Scientific, Loughborough, UK) pre-filled with 250 ml of 100% isopropyl alcohol, which was placed in a −80°C freezer for a minimum of 4 hours to a maximum of 24 hours. Cryotubes were then transferred to a liquid nitrogen storage container.

### Synthetic CSFV peptides

A synthetic overlapping peptide library was designed which comprised pentadecamer peptides off-set by four residues. The peptide sequences were designed using the predicted polyprotein of CSFV C-strain Riems (GenBank accession number AY259122.1). The synthesized library of 945 peptides (JPT Peptide Technologies, Berlin, Germany) was reconstituted in sterile 10 mM HEPES (Life Technologies) buffered 40% acetonitrile (Sigma-Aldrich) at a concentration of 1 mg/ml. For initial screening, peptides were combined into pools representing the structural and non-structural proteins of CSFV and diluted in cRPMI and used at a final total peptide concentration of 1 µg/ml, unless otherwise stated. In order to identify the antigenic peptides from positive pools, a two-way matrix system was adopted to screen peptides representing the non-structural proteins: NS5A, NS3, NS2 and the structural protein E2. Matrix pools were designed so that each peptide was uniquely present in 2 different pools. The 121 NS5A peptides were prepared in 22 peptide pools (A-V), the 110 NS2 peptides in 21 peptide pools (A-U), the 168 NS3 peptides 26 peptide pools (A-Z) and the 90 E2 peptides in 19 matrix peptide pools (A-S). An illustration of how the peptides were combined into matrix pools is shown in Table 1 using NS2 peptides as an example. After identification of the antigenic pentadecamers, truncated 8-11mers of consensus antigenic sequences were designed, synthesised (JPT Peptide Technologies), reconstituted and prepared as described above.

**Table 1:** Example of the two-way matrix-pooling system used to identify T cell reactive peptides from pools representing CSFV proteins.

		NS2 Matrix pools*										
		A	B#	C	D	E	F	G	H	I#	J	K
**NS2 Matrix pools contd.**	**L**	1	2	3	4	5	6	7	8	9	10	11
	M	12	13	14	15	16	17	18	19	20	21	22
	N	23	24	25	26	27	28	29	30	31	32	33
	**O** #	**34**	35	36	37	38	39	40	41	**42**	43	44
	**P** #	**45**	46	47	48	49	50	51	52	**53**	54	55
	Q	56	57	58	59	60	61	62	63	64	65	66
	R	67	68	69	70	71	72	73	74	75	76	77
	S	78	79	80	81	82	83	84	85	86	87	88
	T	89	90	91	92	93	94	95	96	97	98	99
	U	100	101	102	103	104	105	106	107	108	109	110

*The 110 overlapping 15mer peptides (#1-110) representing CSFV NS2 were pooled using a matrix system so that each peptide was uniquely represented in two matrix-pools (Pools A-U).

# Matrix pools B, I, O and P stimulated CD8 T cell reactivity from Pig AN7 and so NS2 peptides #35, 42, 46 and 53 were identified for further testing.

### Porcine IFN-γ ELISpot assay

ELISpot plates (96 well Multiscreen-IP Filter Plates; Millipore, Watford, UK) were prepared by pre-wetting each well with 15 µl of 35% ethanol for 1 min then washing 3 times with sterile PBS. The capture antibody (anti-porcine IFN-γ mAb, P2G10, BD Biosciences, Oxford, UK), prepared at 0.5 µg/ml in PBS, was added at 50 µl/well and the plates incubated at 4°C overnight. Capture antibody was then decanted and plates washed 3 times with unsupplemented RPMI-1640 medium. Plates were blocked by addition of 100 µl/well cRPMI and incubation for at least 1 hr at 37°C. Freshly isolated PBMC were suspended at 5×10^6^/ml in cRPMI and 100 µl of cells was added to each well. CSFV Alfort 187 strain was diluted in cRPMI and added at a multiplicity of infection (MOI) of 1. Concanavalin A (Sigma-Aldrich) at 10 µg/ml and mock virus-infected PK-15 cell cryolysate supernatant were used as positive and negative controls, respectively. All conditions were tested in triplicate and plates were incubated at 37°C in a 5% CO_2_ humidified atmosphere for 18 hours. Well contents were discarded and 100 µl of cold water was added to each well and incubated for 5 min. Wells were then washed 5 times with PBS containing 0.05% Tween 20 (ELISpot Wash Buffer). Biotinylated anti-porcine IFN-γ mAb (P2C11, BD Biosciences, Oxford, UK), diluted to 0.167 µg/ml in PBS, 0.05% Tween 20, 1% FBS was added (50 µl/well) and plates incubated at 4°C overnight. Plates were washed 3 times with ELISpot Wash Buffer and incubated with streptavidin-HRP (R&D Systems, Abingdon, UK; 0.5 µg/ml, 50 µl/well) for 1 hr at 37°C. After plates were washed 5 times, BCIP/NBT substrate (R&D Systems, Abingdon, UK, 100 µl/well) was added and plates incubated at RT in the dark until spots became visible, typically 15–60 min. The substrate was then discarded and the plates washed extensively with water and left to dry in the dark. Spots were visualized using an automated ELISpot reader (AutoImmun Diagnostika, Straβberg, Germany).

### Multi-parameter cytofluorometric analysis of PBMC responses

Both freshly isolated and cryopreserved PBMC were used in this study. To resuscitate cryopreserved cells, cryovials were rapidly thawed in a 37°C water bath and cells transferred to tubes containing 10 ml of pre-warmed (37°C) cRPMI. Cells were washed by centrifugation, 930×*g* for 5 minutes at RT, and resuspended in fresh warm cRPMI. Cell densities were calculated using flow cytometry as described above and adjusted to 1×10^6^ cells/ml and 100 µl transferred to wells of a 96-well round bottom microtitre plate (Costar, Fisher Scientific). PBMC were stimulated with 100 µl of CSFV Alfort-187 strain, at a MOI = 1, or with 100 µl of peptide pools or individual peptides at 1 µg/ml, unless otherwise stated. Mock-virus PK15 supernatants/cRPMI medium and ConA (10 µg/ml) were used as negative and positive controls, respectively. Cells were incubated at 37°C for 2 (peptide-stimulation) or 14–16 (virus-stimulation) hours and then brefeldin A (GolgiPlug, BD Biosciences) was added (0.2 µl/well) and cells were further incubated for a further 16–18 or 6 hours following stimulation with peptide or CSFV, respectively. To detect cytotoxic degranulation, CD107a-Alexa Fluor 647 or IgG1 isotype control-Alexa Fluor 647 mAbs (both AbD Serotec, Oxford, UK; 10 µl/well) and monensin (Golgi Stop, BD Biosciences; 0.2 µl/well) were added in conjunction with Brefeldin A.

Cells were washed in Dulbecco's PBS without Mg^2+^ and Ca^2+^ (DPBS; Life Technologies) and stained with Near Infra-Red Fixable Live/Dead Viability Dye (Life Technologies) for 30 minutes at 4°C. Cells were washed twice with DPBS supplemented with 2% FBS and 0.09% sodium azide (FACS buffer) and stained with mAbs specific for surface markers for 10 min at RT: CD8α-PE (76-2-11, BD Biosciences) and CD4-PerCP-Cy5.5 (74-12-4, BD Biosciences), CD25 (K231.3B2, AbD Serotec) and CD27 (b30c7, kindly provided by Dr Wilhelm Gerner, University of Veterinary Medicine, Vienna, Austria [23]). Cells were washed twice with FACS buffer and staining with CD25 and CD27 was visualized by incubation of cells with APC-conjugated rat anti-mouse IgG1 (BD Biosciences) for 10 min at RT and then washed twice with FACS buffer. Surface stained cells were fixed and permeabilised using CytoFix/CytoPerm Solution (BD Bioscience) for 20 min at 4°C. After two washes in BD Perm/Wash Buffer (BD Biosciences), PBMC were incubated with cytokine specific mAbs at RT for 10 minutes in the dark. mAbs used for cytokine staining were: IFN-γ-FITC or –Alexa Fluor 647 (CC302, AbD Serotec), TNF-α-Pacific Blue (MAb11, Biolegend, Cambridge BioScience, Cambridge, UK) and IL-2 (A150D 3F1, Life Technologies), labelled using Zenon Alexa Fluor 647 mouse IgG2a labelling kit (Life Technologies). IgG1-FITC or –Alexa Fluor 647 isotype control mAbs were used to control staining with IFN-γ mAbs. Un-stained cells were used as control for IL-2 and TNF-α. The cells were given two final washes in BD Perm/Wash buffer and re-suspended in FACS buffer prior to flow cytometric analysis on a MACSQuant Analyzer (Miltenyi Biotec) or CyAn ADP (Beckman Coulter, High Wycombe, UK) flow cytometers. Cells were analyzed by exclusion of doublets, followed by gating on viable cells (Live/Dead Fixable Dead Cell Stain negative) in the lymphocyte population and defined lymphocyte subpopulations were then gated upon and their expression of cytokines assessed. Gates were set using the corresponding isotype/unstained controls and values were corrected by subtraction of the % positive events in the biological negative control (cRPMI or mock-virus supernatant stimulated). The number of singlet live lymphocytes acquired for analysis ranged from 200,000–400,000, which translated to 20,000–80,000 CD8 T cells being analysed.

### Sequence analysis of T cell antigenic peptides/epitopes

The sequences of the identified T cell antigenic regions/epitopes were aligned against the predicted full-length polyprotein sequences of 14 CSFV isolates (GenBank accession numbers shown in parentheses): Genotype 1.1 - Brescia (AF091661), Alfort/187 (X87939.1), KC Vaccine (AF099102), ALD (D49532), GPE^−^ (D49533), Alfort-A19 (U90951), cF114 (AF333000), Shimen (AF092448), Koslov (HM237795) and SWH (DQ127910); Genotype 2.1 - Penevezys (HQ148063); Genotype 2.3 - Borken (GU233731) and Alfort Tübingen (AAA43844); Genotype 3.4 - 94.4/IL/94/TWN (AY646427.1); the BVDV reference strain NADL (AJ133738) and the border disease virus (BDV) reference strain BD31 (U70263), using the *clustal W* algorithm on MegAlign (DNAStar Lasergene 9 Core Suite, Madison, WI, USA).

The sequences of the identified T cell antigenic regions/epitopes were also aligned with the corresponding sequences of the CSFV isolates UK2000/7.1 [18] and CBR/93 [19]. These sequences were generated using RNA from CSFV strains UK2000/7.1 and CBR/93 and creating cDNA by reverse transcription as previously described [22]. A 5 µl aliquot of cDNA was used as template for PCR amplification with high fidelity Platinum Taq in a 45 µl reaction mix containing: 1 µl dNTP (10 mM of each dNTP, Promega, Southampton, UK), 5 µl 10x PCR buffer, 1.5 µl MgSO_4_ (50 mM), 0.2 µl Platinum Taq (5 U/ml) (all Life Technologies) and 0.5 µl of each 20 µM primer solution (Sigma-Aldrich). The following primers (5′-3′) were used to amplify the region encoding the viral proteins Npro, Core and Erns: HE5 TGGGAGTGGAGGAACCG and HE ErnsR TCNGGGGCGAAGTCAGACA. Sequencing was achieved with these primers together with HE2 GTGATGGGRGTACGA CCTGATAGG, Core-F-954 AGAGCATGAGAAGGACAGYA, Core-R-1211 GTGCCRTTGTCACTYAGGTT, HE15 ErnsR internal GTGTACCATATATACCCTATT, HE16 ErnsR internal GTCTATGTTGTACCAGTTGC and HE ErnsF CAAGAYGGCCTGTACCAYAAY. The other viral coding regions were amplified and sequenced with the same primer pairs, specifically primers used for E2 were E2-F-2846 GTCRTAGAGTGCACRGCAGT and E2-R-3613 GTGTGGGTRATTAAGTTCCCTA, for NS2 were NS2-F-3840 TAGTAGTCGYYGTGATGTTR and NS2-R-4248 GCCCACATCGTAAAMACCA, for NS3 were NS3-F-5974 AGGATAGGKGAGATGAAGG and NS3-R-6332 TTCATCTCCTCTACTGGTATC and for NS5A were NS5A-F-9278 ACTATGACTACAGGGGAG and NS5A-R-9857 GATATCTTTGTGGAGTCTGT. The following thermal profile was adopted: 94°C for 2 minutes, 40× (94°C 30 seconds; 53°C 30 seconds for E2/49°C 30 seconds for core and NS3/46°C 30 seconds for NS2 and NS5A; 72°C 60 seconds); 72°C 1 minute and a 4°C hold. Sequencing reactions using purified amplicons were set up in both directions using standard Sanger sequencing (Central Sequencing Unit, AHVLA, Addlestone, UK) the amino acid sequences deduced from the assembled amplicon sequences were aligned with the matching antigenic peptide.

### MHC class I haplotype determination by low-resolution (Lr) PCR-based analysis

Pigs used in this study were genotyped for their swine leukocyte antigen (SLA) class I haplotypes by low-resolution PCR screening assays (PCR-SSP) on PBMC-derived genomic DNA as previously described [24].

### Data analysis and statistics

Graphical and statistical analysis was performed using GraphPad Prism 5.04 (GraphPad Software Inc, La Jolla, USA). Data was represented as means with standard error of means (SEM) quoted to indicate the uncertainty around the estimate of the group mean. A two-tailed unpaired t-test or a one-way-analysis-of-variance (ANOVA) followed by a Dunnett's multiple comparison test was used and a p-value <0.05 was considered statistically significant.

### Nucleotide and predicted amino acid sequence GenBank accession numbers

The nucleotide and predicted amino acid sequences from CSFV UK2000/7.1 and CBR/93 which include the identified antigenic peptides/epitopes have been deposited in GenBank under the following accession numbers: CSFV UK2000/7.1 NS2 (KF771869), NS3 (KF771871) and NS5A (KF771873) and CSFV CBR/93 core (KF784898), E2 (KF771867), NS2 (KF771868), NS3 (KF771870) and NS5A (KF771872). The following existing submissions displayed 100% identity with sequenced regions for CSFV UK2000/7.1 core (AFJ79223) and E2 (JQ411582),

## Results

### Detection of CSFV-specific CD8 T cell responses following vaccination and challenge

**Figure 1:**
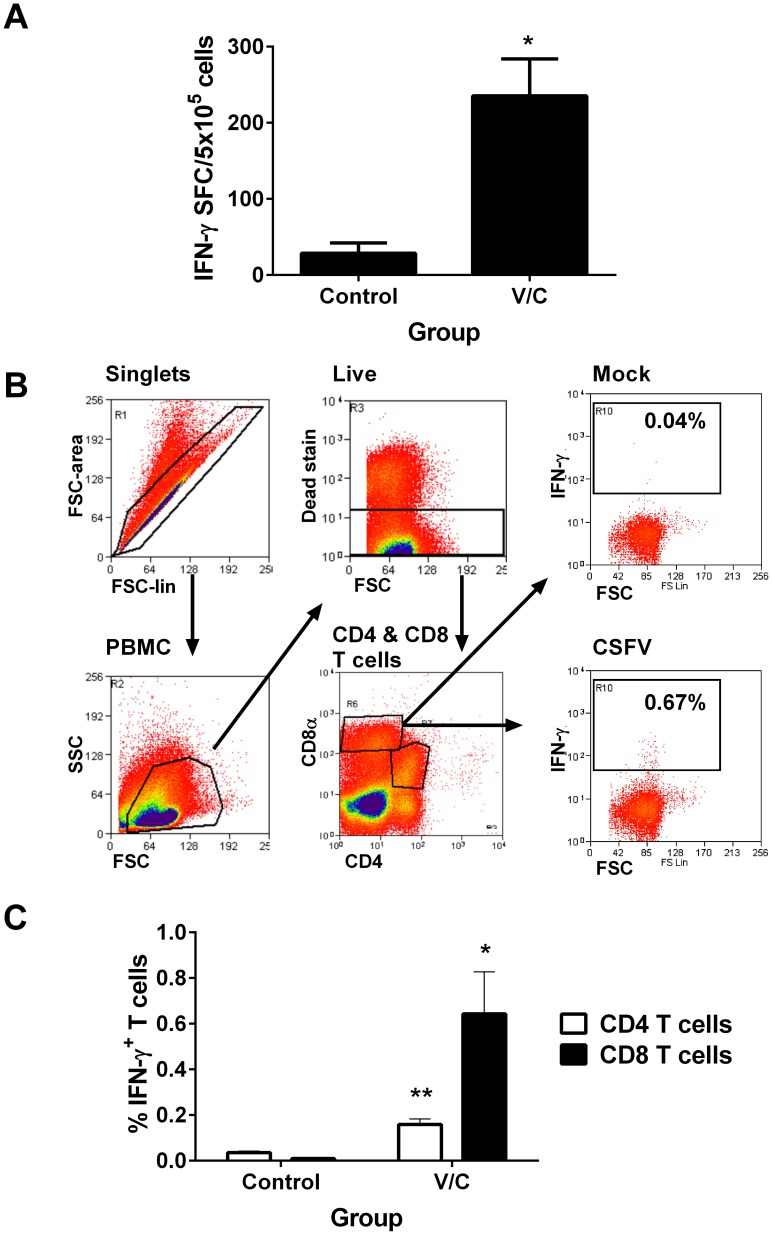
Vaccination with C-strain and challenge with virulent CSFV induces predominately a virus-specific IFN-γ CD8 T cell response. Pigs were vaccinated on day -5 and then challenged with CSFV Brescia strain on days 0 and 28 post-challenge. Seven days after the second challenge, PBMC from vaccinated/challenged (V/C) and control animals stimulated with CSFV and IFN-γ release measured by ELISpot assay. Panel A shows the mean mock-virus stimulated corrected IFN-γ spot-forming cells (SFC)/5×10^5^ PBMC for each group and error bars represent SEM. Panel B shows the gating strategy used to interrogate responses in singlet, live CD8 T cells (CD8α^high^CD4^-^) and memory CD4 T cells (CD8α^low^CD4^+^) [25]. Representative dot plots show the IFN-γ staining of singlet, live CD8 T cells following stimulation with mock-virus or CSFV. Panel C shows the mean mock-virus stimulated corrected % IFN-γ expressing memory CD4 T cells and CD8 T cells +/− SEM. for 8 V/C animals and 3 control animals. Values of control and V/C groups were compared using a two-tailed un-paired t-test and significance is indicated by **p<0.01, *p<0.05.

Animals vaccinated in Experiment 1 were solidly protected against both challenge infections, at 5 and a further 28 days post vaccination, with no evidence of leukopenia or clinical signs of the disease being observed (data not shown [21]). One week after the second challenge inoculation a significant IFN-γ response following *in vitro* stimulation with CSFV was observed in PBMC from vaccinated/challenged (V/C) animals, but not control pigs (Fig. 1A). Using the flow cytometric gating strategy displayed in Fig. 1B, CSFV-specific IFN-γ expression was detected in the CD4^−^CD8α^high^ and CD4^+^CD8α^low^ populations, which in swine have been proposed to represent CD8 and activated/memory CD4 T cell populations respectively [25]. CSFV-specific IFN-γ responses were detected in PBMC from V/C animals, but not control pigs, with the CD8 T cell population dominating the IFN-γ response (Fig. 1C). From the 8 V/C pigs, the greatest CD8 T cell response was identified in pigs AN5, AN7, AN11 and AN13, and these animals were selected for an in-depth analysis of the specificity of their CD8 T cell responses.

### Screening of a CSFV proteome-wide peptide library to identify CD8 T cell antigens

**Figure 2:**
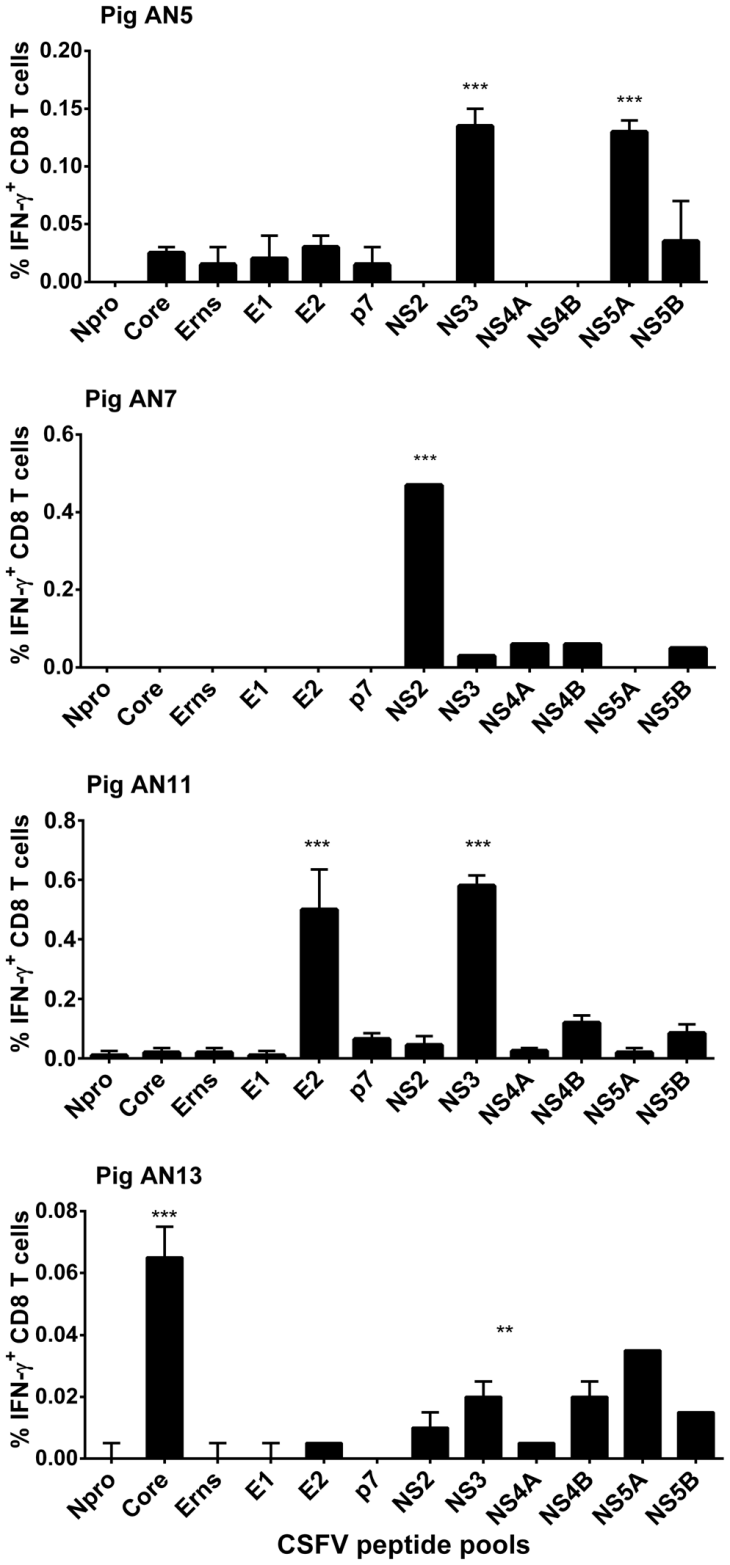
CD8 T cells from pigs vaccinated with C-strain and challenged with virulent CSFV display distinct profiles of antigen reactivity. Fourteen days after re-challenge with CSFV Brescia strain, PBMC from four selected pigs were stimulated with synthetic peptide pools representing the 12 CSFV proteins and IFN-γ expression was assessed by flow cytometry. Graphs show the mean unstimulated corrected % IFN-γ^+^ CD8 T cells for each animal in response to peptide pools +/− SEM. Statistical analyses were performed using a one-way ANOVA followed by a Dunnett's multiple comparison test versus the unstimulated cells; ***p<0.001, **p<0.01.

To identify CSFV T cell antigens, PBMC from the four selected V/C pigs were stimulated *in vitro* with pools of overlapping 15mer peptides representing the 12 CSFV proteins and CD8 T cell reactivity was screened using IFN-γ detection by flow cytometry (Fig. 2). Significant IFN-γ responses were observed in all V/C animals and each animal reacted against a unique profile of antigens. Pig AN5 mounted a significant IFN-γ response against NS3 and NS5A peptides, pig AN7 reacted significantly against the NS2 peptide pool, pig AN11 responded to peptides representing the E2 and NS3 proteins and pig AN13 mounted the greatest response to the core peptides with significant reactivity also observed against NS5A.

### Identification of CD8 T cell epitopes on CSFV

**Figure 3:**
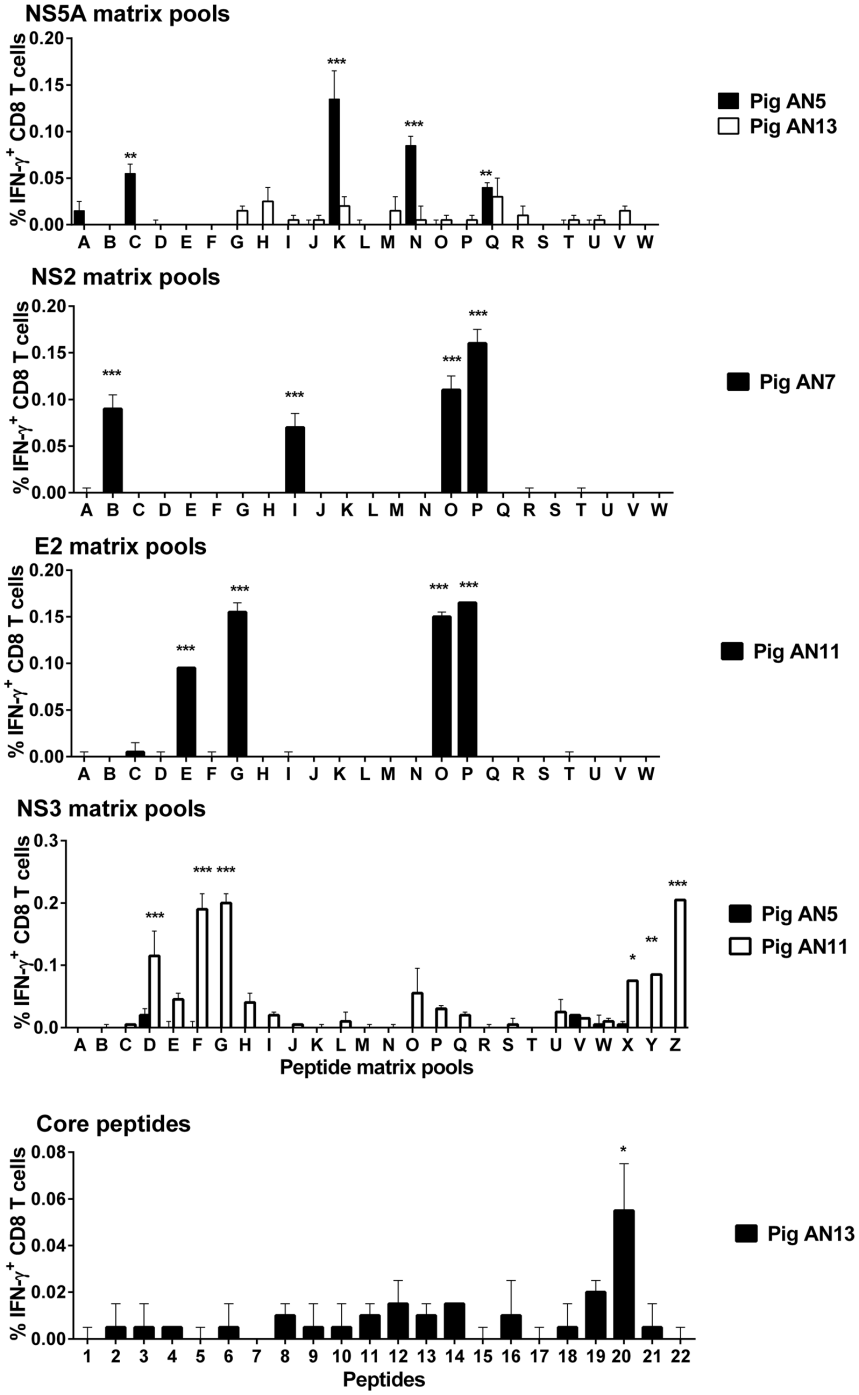
Identification of putative antigenic peptides recognised by CSFV specific CD8 T cells. Twenty one days after re-challenge, PBMC from pigs AN5, AN7, AN11 and AN13 were stimulated with synthetic peptides pooled in a 2-way matrix for proteins E2 (pig AN11), NS2 (pig AN7), NS3 (pigs AN5 and AN11) and NS5A (pigs AN5 and AN13). Peptides representing the core protein were screened individually (pig AN13). IFN-γ expression by CD8 T cells was assessed by flow cytometry as described above. The mean unstimulated corrected % IFN-γ expressing CD8 T cells are presented and error bars represent SEM. Statistical analyses were performed using a one-way ANOVA followed by a Dunnett's multiple comparison test versus unstimulated cells; ***p<0.001, **p<0.01, *p<0.05.

To identify the peptide targets of these CD8 T cell IFN-γ responses, a two way matrix system was adopted to screen peptides representing the non-structural proteins NS5A, NS3, NS2 and the structural protein E2, whereas the peptides spanning the core protein were screened individually. The matrix pools were designed so that each peptide was uniquely present in 2 defined pools. An illustration of how peptides were combined into matrix pools and how the CD8 T cell IFN-γ responses to these pools enabled the identification of putative antigenic peptides is shown in Table 1, using NS2 peptides as an example. PBMC from pigs AN5 and AN13 were stimulated *in vitro* with the NS5A matrix peptide pools and IFN-γ^+^ CD8 T cells were identified using flow cytometry. Pools C, K, N and Q induced a significant IFN-γ response in CD8 T cells from pig AN5 (Fig. 3). By analysing the peptide constituents of the reacting matrix pools, four potential antigenic 15mers were identified for further testing: peptides #25, 33, 58 and 66. Pig AN13 did not mount a significant CD8 T cell IFN-γ response to any of the NS5A matrix pools. PBMC from pig AN7 were stimulated with the NS2 matrix peptide pools and a CD8 T cell IFN-γ response was observed against pools B, I, O, P (Fig. 3) identifying 4 putative antigenic peptides: #35 42, 46 and 53. PBMC from pigs AN5 and AN11 were stimulated *in vitro* with the NS3 matrix peptide pools and peptides present in the pools D, F, G, X, Y and Z induced a statistically significant CD8 T cell IFN-γ response, which indicated that peptides #134, 136, 137, 147, 149, 150, 160, 162 and 163 were potentially antigenic. Pig AN5 did not mount a significant response to the NS3 matrix pools. E2 matrix peptide pools were screened with PBMC from pig AN11 leading to the identification of significant reactivity against pools E, G, O and P; suggesting reactivity against peptides 45, 47, 55 and 57.

The identified putative antigenic peptides were next screened individually to assess their recognition by CD8 T cells. PBMC from pig AN5 were found to show significant reactivity to overlapping NS5A peptides 33 and 58, but not 25 and 66, suggesting that a single epitope lay in the 11mer consensus region LSRVDNALLKF (data not shown). Of the four possible antigenic NS2 peptides, we observed that the overlapping peptides 42 and 46, with the consensus sequence LISTVTGIFLI, but not 35 and 53, induced a statistically significant greater number of IFN-γ expressing CD8 T cells from pig AN7 compared to un-stimulated controls (data not shown). PBMC from pig AN11 showed significant reactivity against two pairs of overlapping peptides, E2 peptides 47 and 55 and NS3 peptides 134 and 163 with 11mer consensus sequences of RYYEPRDSYFQ and VEYSFIFLDEY, respectively (data not shown). Due to its short length, peptides spanning the core protein were screened individually using PBMC from pig AN13. Significant CD8 T cell IFN-γ responses were observed against the 15mer peptide #20 (PESRKKLEKALLAWA) (Fig. 3).

**Figure 4:**
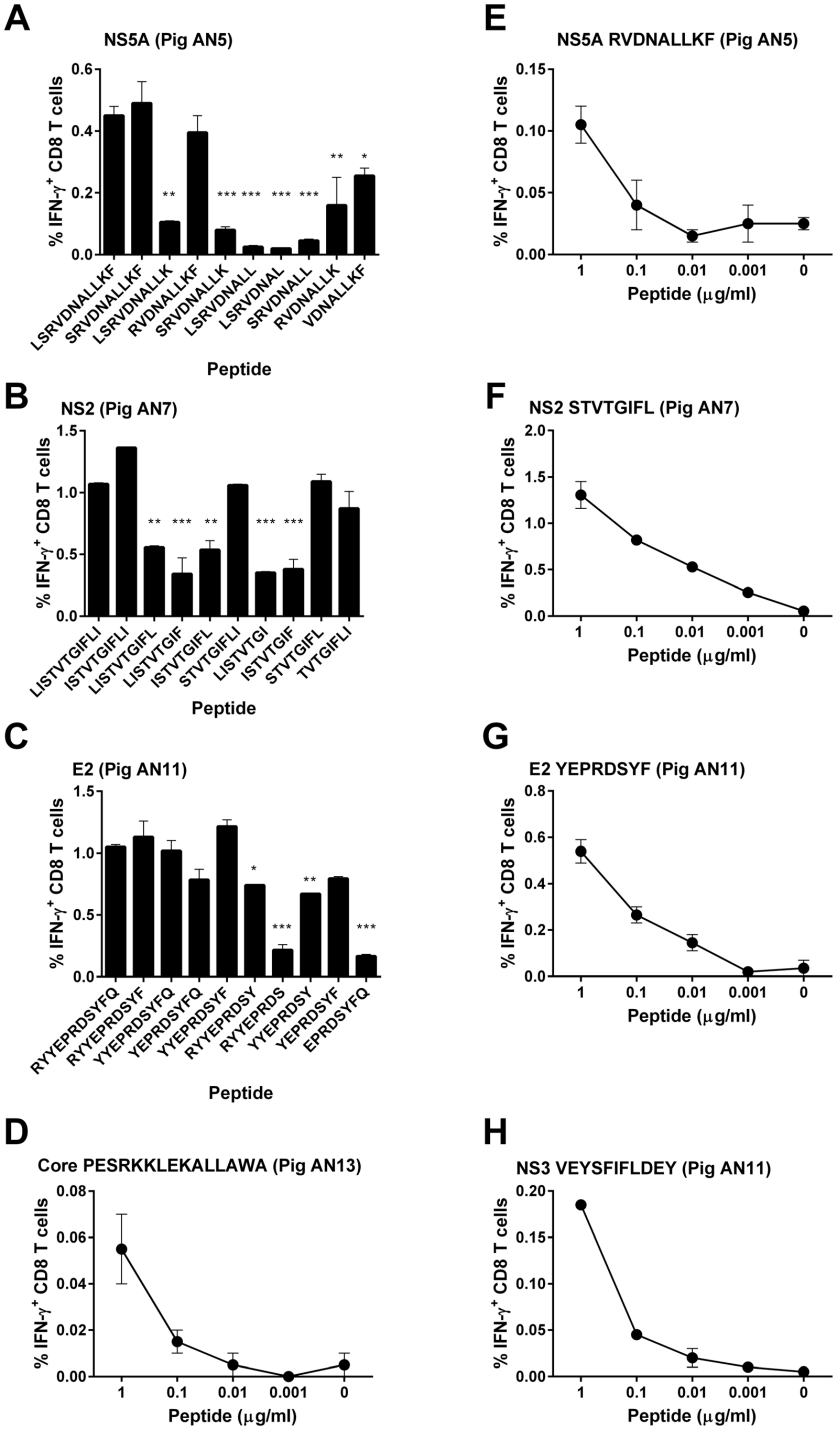
Identification of minimal length antigenic peptides recognised by CSFV specific CD8 T cells. PBMC from pigs AN5, AN7, AN11 and AN13, collected at day 28 or cryopreserved at later time-points were stimulated with the identified 15mer or consensus 11mer antigenic peptides and the truncated derivatives of NS5A LSRVDNALLKF, NS2 LISTVTGIFLI and E2 RYYEPRDSYFQ and IFN-γ expression by CD8 T cells assessed by flow cytometry. Panels A, B and C shows the mean unstimulated corrected % IFN-γ expressing CD8 T cells +/− SEM. Statistical analyses were performed using a one-way ANOVA followed by a Dunnett's multiple comparison test versus the previously identified 11mer antigenic peptide; ***p<0.001, **p<0.01, *p<0.05. Panels D-H show reactivity against a log_10_ dilution series of the identified minimal length antigenic peptides (NS5A RVDNALLKF, NS2 STVTGIFL and E2 YEPRDSYF) or antigenic regions (core PESRKKLEKALLAWA and NS3 VEYSFIFLDEY).

The length of CD8 T cell epitopes can vary between 8 and 11 amino acids. In order to identify the minimal length antigenic peptides and define the natural epitopes, we synthesised the two possible 10mer, three 9mer and four 8mer sequences for each consensus 11mer sequence of overlapping peptide pairs (NS5A-LSRVDNALLKF NS2-LISTVTGIFLI, E2-RYYEPRDSYFQ). Due to a limitation in the numbers of PBMC cryopreserved from pigs AN13 and AN11, we were unable to identify the minimal length antigenic peptides for NS3_1902–1912_ VEYSFIFLDEY (pig AN11) and core_241–255_ PESRKKLEKALLAWA (pig AN13) regions. Following stimulation of PBMC from pig AN5 with the NS5A 11mer LSRVDNALLKF and truncated derivatives (Fig. 4A), one 10mer, two 9mers and the four 8mers induced a statistically significant lower number of IFN-γ producing CD8 T cells than the 11mer, suggesting that the 9mer RVDNALLKF was the minimal length antigenic peptide. Similar analysis with pig AN7 (Fig. 4B) identified the minimal length antigenic peptide on NS2 to be the 8mer STVTGIFL. A 8mer, YEPRDSYF, located on E2, was identified as being the shortest peptide recognised by CD8 T cells from pig AN11 (Fig. 4C). Responses to all antigenic peptides were all shown to be dose-dependent and the threshold concentration at which responses became undetectable was proportional to the frequency of the peptide specific T cell population (Fig. 4D–H).

### The sequences of CD8 T cell epitopes are well conserved among CSFV strains

Using the Clustal W protein alignment tool, we investigated the conservation of the identified CD8 T cell epitopes/antigenic regions among different CSFV strains (Table 2). We observed that the antigenic region PESRKKLEKALLAWA, located on core protein, showed only one amino acid substitution in the genotype 3.3 strain CBR/93. The NS3 11mer VEYSFIFLDEY displayed one amino acid substitution in the genotype 2.1 strains Penevezys and UK2000/7.1. The E2 epitope YEPRDSYF was 100% conserved across all the CSFV strains analysed. The NS2 epitope STVTGIFL was conserved in all the CSFV strains analysed except the Penevezys strain, where there was a single amino acid substitution. The NS5A epitope RVDNALLKF was conserved among all genotype 1.1 strains tested except Shimen, where a single amino acid substitution was observed. This amino acid substitution was observed in the genotype 2.1 UK2000/7.1 strain together with a second substitution, which was present in the other genotype 2 strains analysed, and 2–3 substitutions were found in the two genotype 3 strains. When compared to the reference BVDV and BDV strains, the antigenic regions/epitopes on core, NS3 or E2 showed no or single amino acid substitutions, 2–3 substitutions were observed in the NS2 epitope and the NS5A epitope was poorly conserved with only three amino acids being shared (Table 2).

**Table 2:** Conservation of identified CD8 T cell epitopes/antigenic regions among different CSFV isolates and the related pestiviruses, bovine viral diarrhoea virus (BVDV) and border disease virus (BDV).

Virus	Strain	Genotype	GenBank	core_241–255_	NS3_1902–1912_	E2_996–1003_	NS2_1223–1230_	NS5A_3070–3078_
CSFV	C-strain Riems	1.1	AY259122.1	PESRKKLEKALLAWA	VEYSFIFLDEY	YEPRDSYF	STVTGIFL	RVDNALLKF
CSFV	Brescia	1.1	AF091661	...............	...........	........	........	.........
CSFV	Alfort/187	1.1	X87939.1	...............	...........	........	........	.........
CSFV	KC Vaccine	1.1	AF099102	...............	...........	........	........	.........
CSFV	ALD	1.1	D49532	...............	...........	........	........	.........
CSFV	GPE^−^	1.1	D49533	...............	...........	........	........	.........
CSFV	Alfort-A19	1.1	U90951	...............	...........	........	........	.........
CSFV	cF114	1.1	AF333000	...............	...........	........	........	.........
CSFV	Shimen	1.1	AF092448	...............	...........	........	........	K........
CSFV	Koslov	1.1	HM237795	...............	...........	........	........	.........
CSFV	SWH	1.1	DQ127910	...............	...........	........	........	.........
CSFV	Penevezys	2.1	HQ148063	...............	....Y......	........	.......M	...T.....
CSFV	UK2000/7.1	2.1	*	...............	....Y......	........	........	K..T.....
CSFV	Borken	2.3	GU233731	...............	...........	........	........	...T.....
CSFV	Alfort Tübingen	2.3	AA43844	...............	...........	........	........	...T.....
CSFV	CBR/93	3.3	#	L..............	...........	........	........	K..DT....
CSFV	94.4/IL/94/TWN	3.4	AY646427	...............	...........	........	........	K..K.....
BVDV	NADL	1a	AJ133738	Q..............	....Y......	F.......	.L.S.V..	D..PE.SEM
BDV	BD31	1a	U70263	...............	....Y......	.....N..	.I.S....	D.EQD..EY

*CSFV UK2000/7.1: core and E2 (JQ411582), NS2 (KF771869), NS3 (KF771871) and NS5A (KF771873).

# CSFV CBR/93: core (KF784898), E2 (KF771867), NS2 (KF771868), NS3 (KF771870) and NS5A (KF771872).

### Differences in CD8 T response specificity is associated with distinct MHC class I haplotype expression

**Figure 5:**
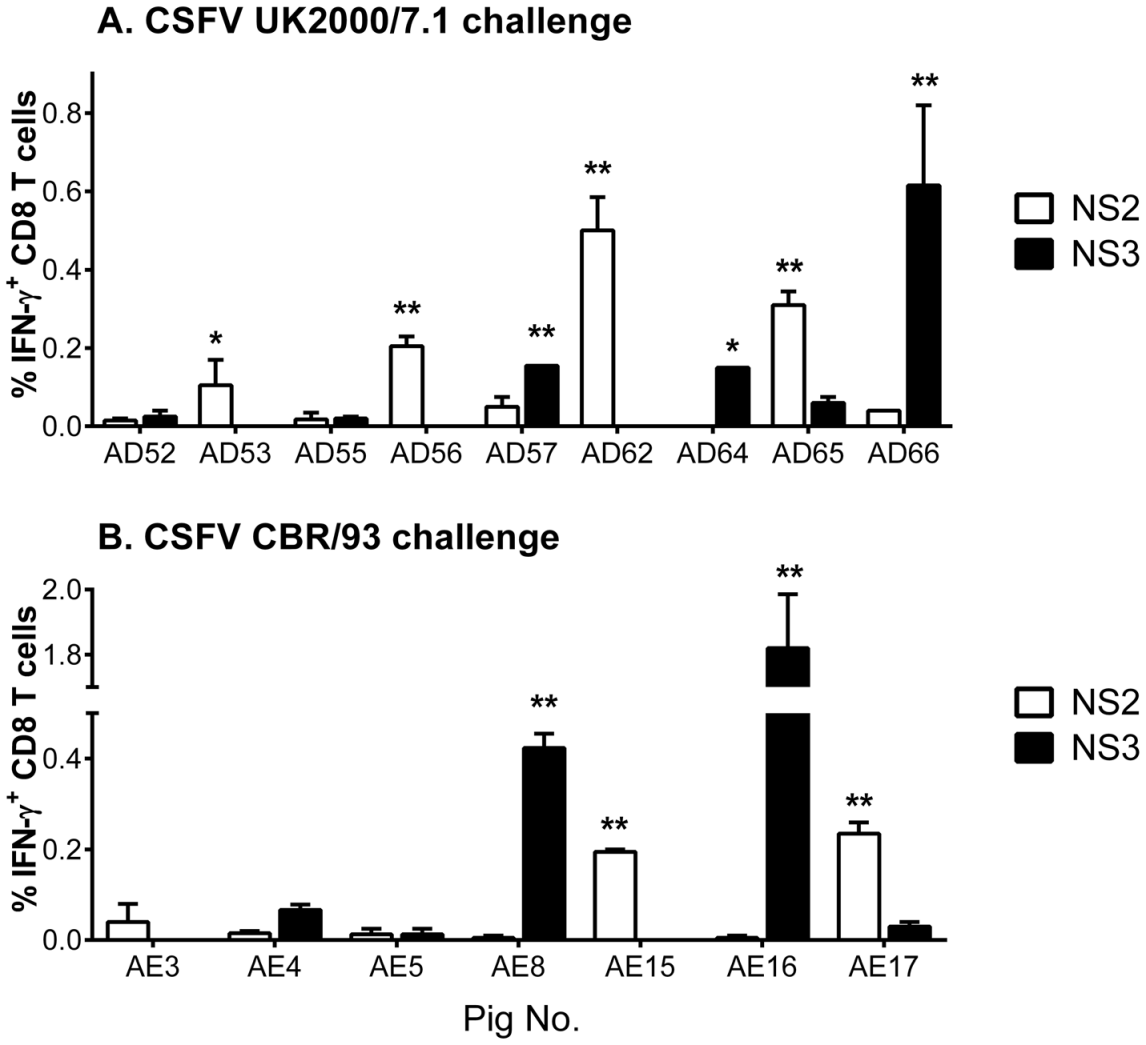
Recognition of the identified epitopes by CD8 T cells from C-strain vaccinated pigs challenged with divergent CSFV strains. PBMC from pigs vaccinated with C-strain CSFV and challenged with CSFV isolate UK2000/7.1 (A) or CBR/93 (B), collected 9 or 12 days post-challenge, were stimulated with antigenic peptides NS2 STVTGIFL and NS3 VEYSFIFLDEY. IFN-γ expression by CD8 T cells assessed by flow cytometry and graphs show the mean unstimulated-corrected % IFN-γ expressing CD8 T cells +/− SEM. Statistical analyses were performed using a one-way ANOVA followed by a Dunnett's multiple comparison test versus the un-stimulated control; **p<0.01, *p<0.05.

We investigated whether the different specificity of CD8 T cell responses between pigs was due to them bearing different MHC class I haplotypes. The porcine swine leukocyte antigen (SLA) class I (SLA-1, SLA-2 and SLA-3) haplotypes of the four pigs were determined using a PCR-SSP-based typing assay. Each animal was heterozygous and no two haplotypes were shared between these animals (Table 3). To assess the recognition of the identified T cell epitopes and antigenic peptides by a larger number of C-strain vaccinated pigs, PBMC collected during Experiment 2 were assayed. Pigs were vaccinated with the C-strain and challenged after 5 or 3 days with the virulent CSFV strain UK2000/7.1 (n = 9) or CBR/93 (n = 7). These animals were solidly protected against challenge with no evidence of leukopenia, viraemia or clinical signs of the disease being observed (as described previously, [5]). Four C-strain vaccinated/UK2000/7.1 challenged pigs (AD53, AD56, AD62 and AD65) and two C-strain vaccinated/CBR/93 challenged pigs (AE15 and AE17) reacted against the NS2 8mer STVTGIFL, with a significantly greater number of IFN-γ expressing CD8 T cells observed compared to unstimulated cells (Fig. 5). A significant CD8 T cell IFN-γ response against the NS3 11mer VEYSFIFLDEY was detected in three C-strain vaccinated/UK2000/7.1 challenged pigs (AD57, AD64 and AD66) and three C-strain vaccinated/CBR/93 challenged pigs (AE4, AE8 and AE16). No responses were observed in these animals against the core, E2 or NS5A peptides (data not shown). To assess whether recognition of the two antigenic peptides was associated with the previously identified MHC class I haplotypes, all animals were typed for SLA class I using the PCR-SSP-based assay (Table 3). No haplotypes were found to be shared between these pigs and the animals used to define the antigenic peptides, but all pigs that reacted against NS2 8mer STVTGIFL displayed the haplotype Lr-22.0 and pigs that reacted against the NS3 11mer VEYSFIFLDEY carried the Lr-01.0 haplotype. Interestingly three NS3 reactor animals also expressed Lr22.0 but no response was detected against NS2. Since we had found that the NS3 11mer VEYSFIFLDEY had a single amino acid substitution in the UK2000/7.1 sequence (a tyrosine at position 4), we assayed responses to both the C-strain and UK2000/7.1 NS3 11mer peptides and found they stimulated comparable CD8 T cell IFN-γ responses in SLA-I Lr-01.0 pigs challenged with CSFV UK2000/7.1 (data not shown).

**Table 3:** SLA class I low-resolution (Lr) haplotypes of C-strain vaccinated pigs and CD8 T cell reactivity against identified antigenic peptides.

Pig n°#	SLA class I haplotype	SLA allele specificity∼			Antigenic peptide
		SLA-1	SLA-2	SLA-3	
AN5	Lr-04.0	04XX	04XX	04XX/hb06	NS5A_3070–3078_
	Lr-24.0	blank	06XX	04XX/hb06	
AN7	Lr-16.0 mod*	11XX	w09XX	06XX	NS2_1223–1230_
	Lr-38.0	15XX	12XX	04XX/hb06	
AN11	Lr-07.0	08XX	05XX	07XX	NS3_1902–1912_ E2_996–1003_
	Lr-28.0	09XX+15XX	05XX	07XX	
AN13	Lr-32.0	07XX	02XX	04XX/hb06	core_241–255_
	Lr-37.0	07XX	w09XX	05XX	
AD53	Lr-22.0	08XX	12XX	06XX(0601)	NS2_1223–1230_
	Lr-37.0	07XX	w09XX	05XX	
AD56	Lr-22.0	08XX	12XX	06XX(0601)	NS2_1223–1230_
	Lr-37.0	07XX	w09XX	05XX	
AD62	Lr-22.0	08XX	12XX	06XX(0601)	NS2_1223–1230_
	Lr-55.0	15XX	es22	04XX/hb06	
AD65	Lr-22.0	08XX	12XX	06XX(0601)	NS2_1223–1230_
	Lr-22.0	08XX	12XX	06XX(0601)	
AD57	Lr-01.0	01XX	01XX	01XX	NS3_1902–1912_
	Lr-37.0	07XX	w09XX	05XX	
AD64	Lr-01.0	01XX	01XX	01XX	NS3_1902–1912_
	Lr-22.0	08XX	12XX	06XX(0601)	
AD66	Lr-01.0	01XX	01XX	01XX	NS3_1902–1912_
	Lr-22.0	08XX	12XX	06XX(0601)	
AD52	Lr-31.0/63.0	15XX	16XX	07XX	
	Lr-36.0	02XX+15XX	11XX	01XX	
AD55	Lr-36.0	02XX+15XX	11XX	01XX	
	Lr-45.0	08XX+cs02	w08XX+10XX	07XX	
AE15	Lr-22.0	08XX	12XX	06XX(0601)	NS2_1223–1230_
	Lr-37.0	07XX	w09XX	05XX	
AE17	Lr-22.0	08XX	12XX	06XX(0601)	NS2_1223–1230_
	Lr-37.0	07XX	w09XX	05XX	
AE04	Lr-01.0	01XX	01XX	01XX	NS3_1902–1912_
	Lr-22.0	08XX	12XX	06XX(0601)	
AE08	Lr-01.0	01XX	01XX	01XX	NS3_1902–1912_
	Lr-01.0	01XX	01XX	01XX	
AE16	Lr-01.0	01XX	01XX	01XX	NS3_1902–1912_
	Lr-55.0	15XX	es22	04XX/hb06	
AE03	Lr-53.0	blank	es22/15XX	08XX	
	Lr-55.0	15XX	es22	04XX/hb06	
AE05	Lr-31.0/63.0	15XX	16XX	07XX	
	Lr-31.0/63.0	15XX	16XX	07XX	

# C-strain vaccinated pigs were challenged with CSFV Brescia (AN), UK2000/7.1 (AD) and CBR/93 (AE).

∼ Medium-/high-resolution specificity not determined.

*16.0 mod.: SLA-1*11XX instead of SLA-1*04XX.

### Functional and phenotypic characterization of CSFV peptide-specific CD8 T cells

**Figure 6:**
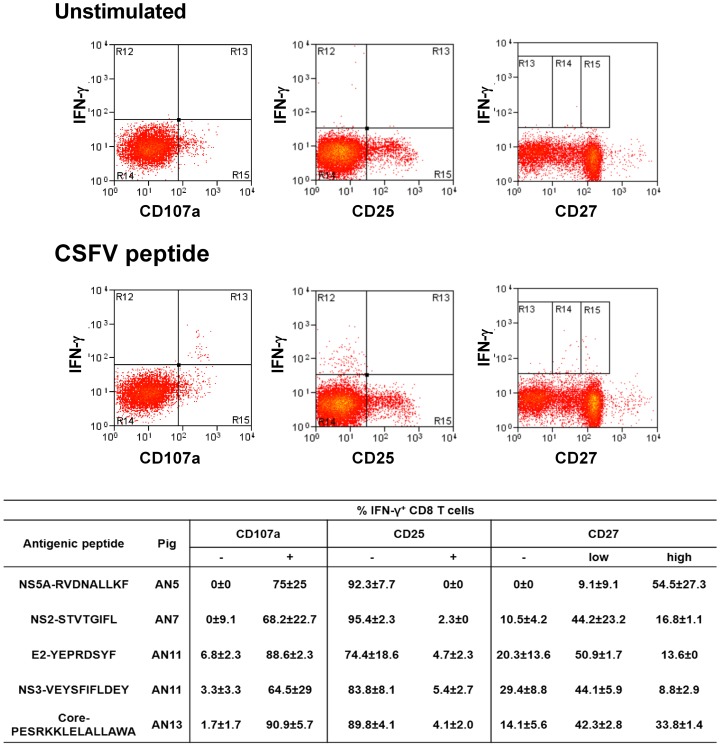
Phenotypic characterization of CSFV epitope-specific CD8 T cells. Cryopreserved PBMC from pigs AN5, AN7, AN11 and AN13 were stimulated with the identified antigenic peptides and the phenotype of IFN-γ expressing CD8 T cells determined by flow cytometry. Dot plots shows representative data of the expression of IFN-γ versus CD107a, CD25 and CD27 by singlet, live CD8 T cells cultures in the presence or absence of CSFV antigenic peptides. The table reports the mean unstimulated-corrected % expression of these markers by peptide-specific IFN-γ^+^ CD8 T cells from triplicate cultures +/− SEM.

**Figure 7:**
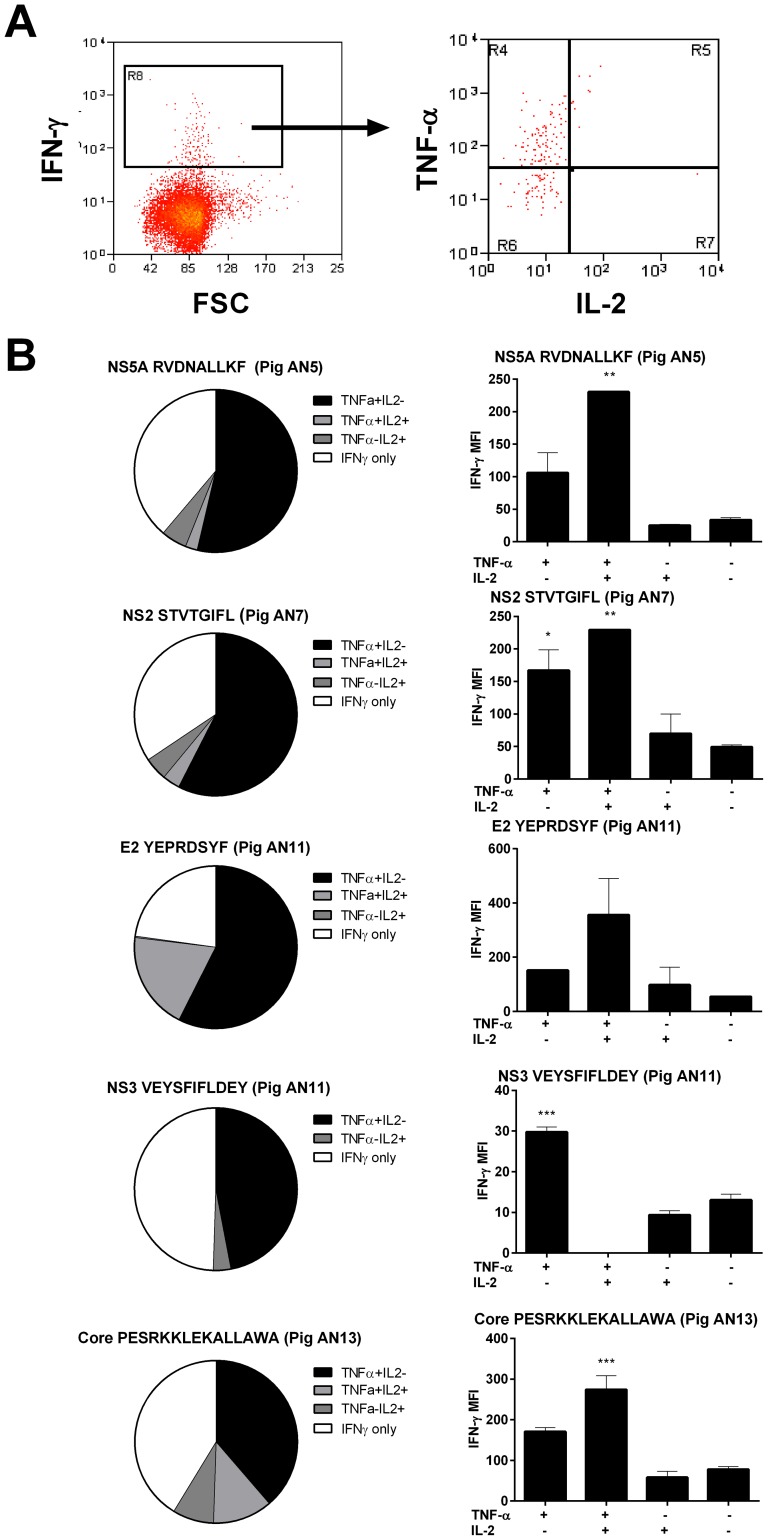
Characterization of polyfunctional cytokine expression by CSFV epitope-specific CD8 T cells. Cryopreserved PBMC from pigs AN5, AN7, AN11 and AN13 were stimulated with the identified antigenic peptides and the expression of IFN-γ, TNF-α and IL-2 by CD8 T cells was simultaneously assessed by flow cytometry. Panel A shows representative dot plots of the expression of TNF-α and IL-2 by peptide specific IFN-γ expressing CD8 T cells and Panel B the relative proportions of IFN-γ^+^ CD8 T cells expressing either of the two other cytokines. In panel C, the mean fluorescence intensity (MFI) of IFN-γ staining in each of the populations is presented. Values represent the mean values for triplicate cultures +/− SEM. Statistical analyses were performed using a one-way ANOVA followed by a Dunnett's multiple comparison test against CD8 T cells expressing only IFN-γ; ***p<0.001, **p<0.01, *p<0.05.

The ability of the five identified antigenic peptides to elicit cytotoxic activity was investigated by assessment of surface mobilisation of CD107a, a marker of degranulation, by IFN-γ^+^ CD8 T cells. Over 90% of IFN-γ^+^ CD8 T cells expressed CD107a on their surface after peptide stimulation, suggesting cytotoxic activity of these cells against the peptide-presenting cells (Fig. 6). With the aim to further characterize the epitope-specific CD8 T cell populations, the expression of the activation markers CD25 and CD27 on IFN-γ^+^ CD8 T cells were investigated using flow cytometry. Interestingly, the majority of peptide-specific IFN-γ^+^ CD8 were CD25^−^ and CD27^+^ (Fig. 6). However, differences were observed in levels of CD27 expression between animals/antigenic peptides with the majority of CD8 T cells that responded to NS5-RDNALLKF expressing high levels of CD27, whereas the others showed the largest proportion of IFN-γ^+^ CD8 T cells in the CD27^low^ population (Fig. 6). We finally analysed the ability of CD8 T cells to produce other cytokines in response to peptide stimulation. The ability of IFN-γ^+^ CD8 T cells to express IL-2 and TNF-α after peptide stimulation was assessed, using the gating strategy illustrated in Fig. 7A. While variability was observed in responses between animals/antigenic peptides, the majority of peptide-specific CD8 T cells expressed either IFN-γ alone or IFN-γ and TNF-α (Fig. 7B). Only a small proportion expressed IFN-γ and IL-2 or all three cytokines, with the largest populations being observed in response to the E2 and core peptides. Interestingly, the cells co-expressing TNF-α or TNF-α/IL-2 expressed incrementally greater amounts of IFN-γ compared to cells which expressed IFN-γ alone or IFN-γ and IL-2 (Fig. 7C), indicative of a greater ‘quality’ of cytokine response.

## Discussion

Despite presenting the porcine immune system with a polyprotein of almost 4000 amino acids in length, this study has shown that the CD8 T cell response to CSFV is highly focussed, dominated by only one or two epitopes each located on five different viral antigens (core, E2, NS2, NS3 and NS5A). Moreover, the specificity of the response varied between animals dependent upon their MHC class I haplotype, which present a high degree of polymorphism in pigs [26]. The antigenic sequences identified in this study showed no overlap with those previously mapped [9], [10]. The most likely reason for this is the MHC haplotype of the animals studied which in the case of the two earlier studies were inbred pigs homozygous for SLA class I haplotype 4a.0. Interestingly, low resolution SLA class I typing suggested that pig AN5 carried haplotype 4.0 and this was confirmed by allele specific PCR (data not shown). It therefore seems likely that the immunodominant response to NS5A was restricted by one of the SLA-1 Lr-24.0 alleles at the expense of responses directed against peptides restricted by the SLA-1 4a.0 alleles. While the underlying mechanisms are unclear, there is evidence that the focussing of CD8 T cell responses toward dominant epitopes can lead to a form of immune suppression of T cells with differing specificities, a process referred to as “immunodomination” [27]. Immunodomination may also explain that, while responses to NS2_1223–1230_ and NS3_1902–1912_ peptides appeared restricted by the Lr-22.0 and 01.0 haplotypes, respectively, animals that were heterozygous for these haplotypes mounted responses solely against NS3. Two of the peptides identified in this study, NS3_1902–1912_ and E2_996–1003_, were homologous to antigenic regions we previously identified using BVDV peptides to screen CSFV-specific T cell responses [6].

To our knowledge this is one of only a few reports on the definition of minimal length antigenic peptides recognised by porcine CD8 T cells. A recent study used the NetMHCpan prediction algorithm [28] to identify a 9mer peptide (MTAHITVPY) from the P1 capsid antigen of the foot-and-mouth disease virus (FMDV) that bound the SLA allele SLA-1*0401 [29], [30]. It was subsequently shown that a SLA-1*0401/MTAHITVPY tetramer stained CD8 T cells from SLA matched FMDV vaccinated pigs and the size of the stained population correlated with cytotoxic responses in these animals [30]. In support of this immunoinformatics approach, the CSFV polyprotein was screened for binding to potential restricting alleles (based on the SLA class I Lr typing results) and two of the peptides NS2_1223–1230_ and NS3_1902–1912_ were predicted by NetMHCpan (www.cbs.dtu.dk/services/NetMHCpan/) to bind strongly to at least one of the class I alleles potentially present in each of the restricting haplotypes: SLA-2*12.01 for NS2_1223–1230_ (potentially present in both haplotypes Lr-38.0 and Lr-22.0) and NS3_1902–1912_ was predicted to strongly bind SLA-1*08.01 (Lr-07-0), SLA-3*07.01 (Lr-28.0);and SLA-2*01.01 and SLA-2*01.02 (Lr-01.0) (data not shown).

Immunodominance in CD8 T cell responses is thought to arise primarily as a consequence of the limitations of peptides to bind with high-affinity to available MHC class I molecules, with additional limitations in antigen processing and the CD8 T cell receptor repertoire also playing a role. Only approximately 1/2000 of the peptides within an antigen can achieve immunodominant status with a given MHC class I allele [31]. Immunodominance is thought to be critical for immunity since numerically prominent CD8 T cells have been shown to confer more effective protection than T cells specific for subdominant epitopes. It has also been shown that the efficacy of peptides in providing protection against a viral challenge is proportional to their binding affinity for the restricting MHC class I molecule [32]. In HIV-1 infection it has been shown that immunodominant CD8 T cell responses may limit virus replication and they are the primary targets of escape mutations [33], [34]. Immunodominance of CD8 T cell responses to Flaviviruses has also been described, with disparity in the degree depending on the virus/host system studied [35], [36]. A recent study, utilising a similar strategy to the present one, assessed the specificity of human T cell response to Dengue virus. Using a cohort of 25 patients, 21 novel CD8 T cell epitopes were identified, with NS3 and NS5 being the most antigenic and the majority of epitopes being recognised by single patients [35].

Immunodominance may also be a key factor in determining the strain specificity of immunity if directed against polymorphic epitopes. The identified epitopes in this study were well conserved amongst CSFV isolates, although for most of these variants it remains to be determined whether these substitutions could affect T cell recognition. NetMHCpan predicted that the mutation of tyrosine in place of a phenylalanine at position 1906 of the NS3 11mer VEYSFIFLDEY would not affect the binding to SLA class I alleles and we were able to show this mutation did not affect T cell reactivity. Moreover, the antigenic core_241–255_ peptide and the E2_996–1003_ and NS3_1902–1912_ epitopes are well conserved between CSFV, BVDV and BDV. Such conserved T cell epitopes could enhance the efficacy of a BVDV/CSFV chimeric vaccine. It has recently been reported that the chimeric vaccine CP7_E2alf induces rapid protection, comparable to the C-strain vaccine [12], [37]. Since this protection precedes the appearance of neutralizing antibodies, it may be that T cell responses against epitopes conserved between BVDV and CSFV are contributing to the protective effect. Support for this hypothesis also stems from our recent studies that showed a close temporal correlation between the induction of CSFV-specific T cell IFN-γ responses and rapid protection induced by the C-strain vaccine [5] and that T cells from these animals cross-react with BVDV derived peptides [6].

Several studies have identified E2 and NS3 as targets of the T cell response against CSFV [6]–[10]. However, our results provide strong evidence that at least in the context of responses induced by C-strain CSFV, E2 and NS3 are not necessarily the major T cell antigens, and other CSFV proteins may be important targets of the CD8 T cell response. These results however may not limit the design of a marker vaccine based on only one or two immunodominant antigens. A study on the related yellow fever virus showed that, in case of abrogation of the dominant CD8 T cell epitope, the frequencies of T cells recognizing the subdominant CD8 T cell epitope increased dramatically [36]. A previous study showed that vaccination with a IFN-α adjuvanted subunit CSFV E2 vaccine fully protected pigs against a challenge administered only 7 days later, suggesting that a T cell response directed solely against E2 may be protective [38]. A recent evaluation of dendrimeric peptide formulations of linear B cell epitopes from E2 and a CD8 T cell antigenic peptide from NS3 induced the production of both neutralizing antibodies and IFN-γ producing cells, which resulted in a degree of protection against CSFV [39]. Significantly, the protection achieved was greatest when the T cell antigenic peptide was included together with the B cell epitopes [40]. Incorporation of T cell epitopes identified in the present study might further enhance the development of such a marker vaccine approach.

We observed that all the identified peptides were able to induce CD107a mobilization in IFN-γ^+^ CD8 T cells. Translocation of lysosomal-associated membrane protein 1 (LAMP1/CD107a) to the cell membrane has been validated as a marker of cytotoxic degranulation by CD8 T and NK cells [41]. Our data support previous studies, which report the ability of the viral antigens E2 and NS3 to elicit cytotoxic activity in vaccinated pigs [7]–[10] and suggest that these T cells possessed cytotoxic activity in addition to cytokine release. We also assessed the polyfunctionality of the peptide-specific CD8 T cell populations, by assessing co-expression of IFN-γ, TNF-α and IL-2, since this may be central to their protective capacity. Studies on HCV have shown that vaccination with vectors expressing NS3 and NS4a proteins induce specific polyfunctional CD8 T cells which are associated with protection [14], [15]. However, the majority of peptide-specific CD8 T cells expressed IFN-γ alone or IFN-γ and TNF-α with only a small percentage co-expressing IFN-γ TNF-α and IL-2. Interestingly, as observed in other systems [42], [43], the subset expressing all three cytokines showed the highest ‘quality’ of response producing more IFN-γ on a per cell basis.

Analysis of activation markers showed that the majority of the CD8 T cells releasing IFN-γ after peptide stimulation did not express the alpha chain of the IL-2 receptor (CD25). In contrast, a previous study reported variable expression of this marker on CSFV-specific IFN-γ CD8 T cells [44]. We speculate that the absence of CD25 expression is not indicative of a naive state of CD8 T cells, but could reflect a memory phenotype of these cells since a previous study reports that after antigen stimulation some human CD8 T cells express high levels of CD25, whereas others display low levels of this marker and the latter preferentially differentiate into long-lived functional memory cells [45]. Similar to what has been described for human CD8 T cell responses to other Flaviviruses, variable levels of expression of CD27 were observed on peptide-specific CD8 T cells [15], [46]. Based on data from human CD8 T cells this may indicate that the majority of CSFV epitope-specific CD8 T cell populations may be T effector memory (T_EM_) cells [47]. Our results suggest that vaccine strategies should be adopted in order to drive the generation of peptide-specific cytotoxic CD8 T_EM_ cells. A peptide-based vaccine targeting dendritic cells to provide a strong signal to naive CD8 T cells should be designed, with the inclusion of cytokines (IL-12 and IFN-γ) or pathogen-recognition receptor agonists as adjuvants.

By utilising a comprehensive approach to determine the antigen specificity of the CD8 T cell response, we have provided strong evidence for immunodominance in the T cell response to CSFV. CD8 T cell responses of individual animals were uniquely focussed on only one or two epitopes which were mapped on the core, E2 and non-structural proteins NS2, NS3 and NS5A. The individual responses were associated with the expression of distinct MHC class I haplotypes and for two of the peptides there was evidence that they are presented by alleles present in other haplotypes. The five identified antigenic peptides were highly conserved across CSFV isolates, and for some were also well conserved when aligned against the other pestiviruses. The responding CD8 T cells displayed cytotoxicity, with the majority of IFN-γ^+^ cells co-expressing the marker CD107a, and populations also releasing TNF-α and/or IL-2. The antigens and epitopes identified and characterised in this study therefore represent attractive vaccine candidates that may be evaluated in the quest to develop the next generation of CSFV vaccines.
